# Individual nutritional intervention for prevention of readmission among geriatric patients—a randomized controlled pilot trial

**DOI:** 10.1186/s40814-021-00926-9

**Published:** 2021-11-15

**Authors:** Mai Østerø Cramon, Ines Raben, Anne Marie Beck, Jens Rikardt Andersen

**Affiliations:** 1 Department of Medicine, Zealand University Hospital, Lykkebækvej 1, 4600 Køge, Denmark; 2 Department of Nutrition, Exercise and Sports, University of Copenhagen, Rolighedsvej 26, 1958 Frederiksberg, Denmark; 3 Institute for Nursing and Nutrition, Faculty of Health, University College Copenhagen, Sigurdsgade 26, 2200 Copenhagen, Denmark; 4 Nutrition Research Unit, Herlev and Gentofte Hospital, Herlev, Denmark

**Keywords:** Readmission, Nutritional Counseling, Home visits after discharge

## Abstract

**Background:**

Approximately 20% of older people are readmitted to the hospital within 30 days of discharge. Even a short hospital stay decreases the ability to cope with the activities of daily living. The aims of this study were to (1) assess the feasibility of recruitment, (2) assess the acceptability of the intervention, and (3) investigate if an individual nutritional intervention could reduce the readmission rate of geriatric patients within 30 days of being discharged to their own homes.

**Methods:**

The unblinded, randomized, controlled pilot trial includes geriatric patients discharged to their own homes. Forty patients were recruited from a medical ward and randomized to standard treatment (*n* = 19) or individualized nutritional intervention (*n* = 21). The intervention was dietary counseling and a nutrition plan before discharge, combined with two home visits performed by an educated nutritionist over a period of 4 weeks. Outcomes were readmission (primary), mortality, protein and energy intake, body weight, activity of daily living, handgrip strength, number of chair stands, and quality of life. Intention-to-treat analysis, per-protocol analysis, and post hoc analysis of readmissions were carried out.

**Results:**

Recruitment was feasible, and there was high compliance to the intervention. There was no difference in readmission between the intervention group and control group 30 days after discharge (29% vs 11%). The individual nutritional intervention had a positive impact on achieving 75% of energy requirements at 30 days for the intervention group compared to the control group (93% vs 47%, *p* = 0.01). No other differences were found between the groups.

**Conclusion:**

The individual nutritional intervention did not prevent readmission among geriatric patients in this trial. Recruitment procedures functioned well, and the intervention was well accepted by the patients.

**Trial registration:**

ClinicalTrial.gov, NCT03519139. Retrospectively registered on 8 May 2018

**Supplementary Information:**

The online version contains supplementary material available at 10.1186/s40814-021-00926-9.

## Key messages regarding feasibility

- *Uncertainties regarding feasibility that existed prior to this study*: It was not known if the geriatric patients were too exhausted to participate in the study and if the geriatric patients would comply with the nutritional intervention. Furthermore, it was not known if the intervention could reduce the incidence rate of readmission.
- *Key feasibility findings from this study*: This study shows that it is feasible to recruit geriatric patients and that there is high compliance to the intervention with a positive effect on nutritional status, but no effect on the readmission rate.
- *Implications of the findings on the design of the main study*: Our results suggest that a full-scale RCT should consist of a multidisciplinary intervention of at least 12 weeks, with a primary outcome of % of readmissions within 6–12 months after discharge from the hospital rather than the readmission rate within 30 days after discharge.

## Introduction

It is well-known that approximately 20% of older patients are readmitted to the hospital within the first 30 days after discharge [1, 2]. Even a short hospital stay decreases the ability to cope with activities of daily living (ADL) [3]. The causes of readmission are complex and might be due to shorter hospitalization time, problems with cross-sectoral transitions, improper medication, or malnutrition at discharge [4]. Malnutrition has serious consequences for older patients, leading to an extended and complicated course of disease and reduction of muscle mass, rehabilitation capacity, and quality of life (QoL) [3, 5–7]. A systematic review showed that 22% of all older hospitalized patients are malnourished and that many older patients are still malnourished at discharge [7]. It is therefore important to ensure that the energy and protein requirements of older patients are met and that their body weight is maintained, on discharge from the hospital. As far as we know, only two RCTs [8, 9], both Danish and both performed in geriatric wards, have investigated the effect of an individual nutritional intervention on the readmission frequency of geriatric patients within 30 days of being discharged to their own homes. One study [9] showed a significant reduction in the readmission rate, and the other [8] did not. These studies exclusively included geriatric patients who were at nutritional risk at the time of screening. The assessment of nutritional risk was based on Nutritional Risk Screening 2002, which does not show if the patients only have protein deficiency [10]. Both these studies showed that nutritional intervention can increase geriatric patients’ intake of energy and protein, but neither reported whether the patients reached their individual requirements. Geriatric patients who are not at nutritional risk but are in protein deficiency—risk losing muscle mass and functional capacity, suffering impaired recovery from illness, and more complications [11–13]. These patients may therefore also be at risk of readmission. In the present study, we found it relevant to include geriatric patients irrespective of their nutritional status. There was no specific focus on their nutritional status during admission to the hospital, so these patients might possibly be more reluctant to take part in a nutritional intervention at discharge. Furthermore, geriatric patients are admitted to several sections of the medical ward at Zealand University Hospital, which might make it difficult to recruit the required number of participants.

The purposes of this pilot study were to (1) assess the feasibility of recruitment, (2) assess the acceptability of the intervention, and (3) investigate if an individual nutritional intervention at discharge could reduce hospital readmission rate in the first 30 days after discharge.

## Methods

### Design

The design was a randomized, controlled, pilot trial without blinding. Research staff and participants were aware of the group allocation. Geriatric patients were randomized using sealed envelopes to either individualized nutritional intervention or standard treatment (no nutritional counseling or home visits). Educated nutritionists performed all procedures. Follow-up was conducted 30 and 60 days after discharge.

### Identification of participants

Participants were recruited from the medical ward at Zealand University Hospital, Koege, Denmark. The medical ward consists of four sections: L1, M1, M2, and M6. L1 is a geriatric section, and M1 is a medical section for chronic obstructive pulmonary disease (COPD) patients, while M2 and M6 are primarily gastroenterological sections. The chief consultant of each section found the geriatric patients via a search of the electronic medical records. Researchers (MC and IR) then had a dialog with the nursing staff concerning the eligibility of each patient in relation to the inclusion/exclusion criteria and whether the patient was fit enough to be visited that day. A visit was postponed if the patient was unwell, confused, or had just received serious information such as notification regarding the severity of their illness. As a rule, patients were contacted the day after admission to the hospital. The nursing staff facilitated access to the patients by briefly presenting the trial and asking whether the patient would like to speak to a trial practitioner. Trial practitioners then visited the patient and informed them orally and in writing about the trial. Patients were given at least 24 h to reflect on their decision and the opportunity to involve a family member or a friend in the subsequent meeting.

### Inclusion criteria

The inclusion criteria were age 65 or older with a minimum of two diagnoses based on the Danish Health Authority description [14, 15], Danish speaking, able to give written informed consent, and able to eat, and planned to be discharged to recovery in their own homes. Patients were excluded if they had cognitive impairment, terminal illness, plans for weight reduction, or scheduled admissions for defined purposes. Patients were assessed as cognitively unable if impairment was stated in their medical records.

### Intervention

The intervention aimed to prevent weight loss and to ensure that patients received at least 75% of their individually estimated energy and protein requirements [16, 17]. The intervention began at the hospital on the day of discharge, and patients in the intervention group received individual nutrition plans and guidance. The nutritionists conducted two visits to the patients’ homes in the course of the 4 weeks subsequent to discharge. Patients received follow-up phone calls between visits, if necessary. The nutrition plan was based on an individual nutritional assessment performed by the nutritionist. The focus was on habitual dietary intake, preferences, food knowledge, and specific problems such as dysphagia, dental status, and nausea. The nutritionist created the individual nutrition plan with a focus on energy and protein-dense food in accordance with the “Recommendations Regarding the Food Served in Danish Institutions” [18], which are the official Danish dietary recommendations for patients with various nutritional diagnoses, including older people and patients at risk of malnutrition [18]. In addition, counseling focused on the size and frequency of main meals and snacks, food and beverages with high energy and protein density, protein quality, and distribution of proteins in meals throughout the day. Patients were also given leaflets with information and recipes for energy- and protein-dense foods and beverages. With an aim to achieving optimal nutritional status, the following factors were considered: prescription of oral nutrition supplements or multivitamins, calcium and vitamin D supplements etc., the need for convenience foods, meals-on-wheels, and contact to relatives and home care providers.

Home visits were planned to take place 3 and 14–21 days after discharge. A 24-h recall interview was conducted at each home visit, in order to evaluate if energy and protein requirements were met, and the patients were weighed. The nutrition plan was revised if the patient had not consumed at least 75% of energy and protein requirements and/or had experienced a weight loss. Patients were encouraged and educated about the importance of meeting their energy and protein requirements and avoiding weight loss. Patients were contacted by phone 1 week after each home visit to discuss how to address any difficulties in complying with the nutrition plan. If relevant, the nutritionist advised the relatives on how to help the patient comply with the nutrition plan.

### Control group

The patients in the control group received standard treatment and were offered nutritional guidance after the last follow-up.

### Data collection/pilot outcomes

Before approaching a patient, the nutritionist screened the patients, in collaboration with the nursing staff, for compliance with the study inclusion criteria. Patients who met the inclusion criteria, agreed to participate, signed a statement of informed consent, and were enrolled in the trial. Baseline data were collected at the hospital on the day of discharge and prior to randomization (Table 1).

**Table 1:** Baseline characteristics of patients collected before randomization at the day of discharge

Group	Intervention, *n* = 21	Control, *n* = 19
Age, years, median (range)	79 (66–92)	74 (65–94)
Female, *n* (%)	11 (52%)	7 (37%)
BMI, kg/m^2^, median (range)	26.6 (21.1–37.2)	26.1 (17.2–34.7)
Living alone^a^, *n* (%)	12 (57%)	6 (32%)
Nutritional risk, ≥ 3 point at secondary screening, *n* (%)	10 (48%)	11 (58%)
Admission diagnosis, *n* (%)		
COPD in exacerbation	7 (33%)	6 (32%)
Infection (UTI, pneumonia without COPD, etc.)	6 (29%)	6 (32%)
Dyspnoea/respiratory failure (without COPD)	4 (19%)	0 (0%)
Pain	0 (0%)	3 (16%)
Diarrhea/gastroenteritis	1 (5%)	2 (11%)
Others	3 (14%)	2 (11%)
Hospital, length of stay, days, median (range)	6 (2–16)	4 (3–29)
Admissions the last year before inclusion, number, median (range)	0 (0–8)	0 (0–4)
Diagnosis, number, median (range)	4 (2–7)	4 (2–10)
Drugs		
Drugs, number, median (range)	11 (4–19)	12 (2–20)
Drugs compliance^a^, good, *n* (%)	21 (100%)	19 (100%)
Nutrition and rehabilitation during hospitalization and status at discharge, *n* (%)		
Dietician during hospitalization	1 (5%)	0 (0%)
ONS prescription issued by medical ward	2 (10%)	2 (11%)
Plan for rehabilitations	6 (29%)	6 (32%)
Dysphagia^a^	0 (0%)	0 (0%)
Nausea, diarrhea, constipation, vomitting^a^	4 (19%)	6 (32%)
Social services, *n* (%)		
Home-services^a,b^	8 (38%)	8 (42%)
Meals-on-wheels^a^	4 (19%)	1 (5%)
Nutritional status		
Weight, kg, median (range)	71.7 (52.4–111.0)	73.1 (48.6–107.4)
Energy requirement, kJ, median (range)	8000 (5700–11,100)	8500 (5400–10,700)
Energy intake, kJ, median (range)	7500 (3500–10,500)	7100 (2600–12,700)
≥ 75% of energy requirement, *n* (%)	13 (62%)	8 (42%)
Protein requirement, g, median (range)	92 (75–122)	99 (69–118)
Protein intake, g, median (range)	61 (18–91)	56 (7–114)
≥ 75% of protein requirement, *n* (%)	6 (29%)	6 (32%)
Muscle strength (handgrip strength (HGS), 30-s chair stand (30-SCS))		
HGS, kg, median (range)	18 (8–44)	19 (9–42)
30-SCS without armrest (WOA), *n* (%)	11(52%)	9 (47%)
30-SCS with armrest (WA), *n* (%)	10 (48%)	8 (42%)
30-SCS not possible to perform, *n* (%)	0 (0%)	2 (11%)
30-SCS (WOA), number of stands, median (range)	7 (4–12)	7 (3–12)
30-SCS (WA), number of stands, median (range)	4.5 (1–6)	6.5 (2–11)
Functional Recovery Score (FRS), median (range)		
FRS total^c^	81 (20–100)	87 (37–100)
Personal activities of daily living	44 (17–44)	44 (28–44)
Instrumental activities of daily living	16 (1–23)	18 (1–23)
Mobility	25 (0–33)	25 (8–33)
Quality of life (QoL), median (range)		
EQ-5D-5L^d^	0.679 (0.183–0.863)	0.660 (–0.011–1)
Visual analog scale (VAS)^e^	50 (30–95)	60 (5–100)

*COPD* chronic obstructive pulmonary disease, *ONS* oral nutritional supplement, *UTI* urinary tract infection

^a^Information from the patients

^b^Four (19%) in the intervention group had home service every day—the rest had primary home service for cleaning twice per month

^c^FRS total score 0–100 (100 is best)

^d^EQ-5D-5L score 1.000 to − 0.624 (1.000 is best)

^e^VAS score 1–100 (100 best health)

The feasibility of recruitment was assessed as the proportion of patients needed for the study who could be included within the pre-defined time frame of the study. Based on former similar studies [8, 9, 19, 20], we expected 50% of the eligible patients to be included. Patient acceptability was assessed as the proportion of patients who completed the planned home visits and the proportion of patients who reached at least 75% of their individually estimated energy and protein requirements.

The study used the National Danish Health Care System definition of readmittance, i.e., “admittance to hospital with the same diagnosis as the original hospitalization.” Readmission frequency was the primary outcome of the study, so patients’ participation in the study was ended if they were readmitted to the hospital. Patients who wished to leave the study before completion were asked for consent to collect data for readmission calculations (date, the cause, and length of the hospital stay). Information on readmission was obtained through electronic medical records.

Nutritional status was assessed by change in weight and achievement of energy and protein requirements. Patients were weighed wearing light indoor clothes and no shoes using project weighing machines (label OBH Nordica Slim light, type 6271), and height was measured using the ward’s fixed altimeter. A 24-h dietary recall interview [21] was conducted in order to estimate the patient’s energy and protein intake. The quantity of food and beverage was assessed based on household measurements, dishes, plates, glasses, and cups used by the patients. Energy and protein content in meals was estimated using the Danish computer program Vitakost [22]. Energy requirement was estimated as basic metabolic rate (BMR), calculated using the Harris-Benedict equation (recommended by the national Danish health authority), and multiplied by physical activity level (PAL). The BMR was multiplied with a stress factor (1.1), if the patient had a chronic disease with inflammation (e.g., chronic obstructive pulmonary disease or cancer), and with a weight increasing factor (1.3), if the patient had a BMI below 18.5 kg/m^2^ [23]. For patients with BMI ≥ 30 kg/m^2^, the energy requirement was estimated by weight multiplied by 100 kJ [16]. PAL was estimated on the basis of the patient’s statement of the level of physical activity (PA) in everyday life from 1.1 (confined to bed) to 1.5 (light PA such as cleaning, shopping, limited physical training every day) [24]. Protein requirement was assessed individually within the range of 1.1–1.5 g/kg body weight/day, depending on the degree of illness, nutritional status, weight loss during hospitalization, body weight, and PAL [25, 26]. Patients with chronic obstructive pulmonary disease were to consume 1.5 g protein/kg body weight/day. Patients with a BMI ≥ 30 kg/m^2^ were required to consume 1.1 g protein/kg body weight/day [16].

Muscle strength was assessed by handgrip strength (HGS) and 30-s chair stand (30-SCS). Handgrip strength was measured using a hydraulic hand dynamometer (SAEHAN, MSD Europe), with the patient seated with the forearm of the dominant hand free at an angle of approximately 90° [27, 28]. The maximum score of the three measurements at 15-s intervals was recorded [27, 28].

Muscle strength in the lower extremity [29] was measured by 30-SCS using project chairs with armrests and a seat height of 46 cm. The patients folded their arms across the chest, then stood up and sat down on the chair as many times as possible for 30 s. A full stand-up was recorded if the patient was more than halfway up after 30 s. If the patient was unable to stand up with arms crossed [29], a modified test with the use of the armrests was applied. A shift from the use of armrest at baseline to no use of armrest at follow-up was recorded as an improvement.

Activity of daily living (ADL) was assessed by Functional Recovery Score (FRS) [30]. The 11-item questionnaire comprises three main components: personal ADL (P-ADL) assessed by four items, instrumental ADL (I-ADL) assessed by six items, and mobility assessed by one item [30]. P-ADL comprised 44%, I-ADL 23%, and mobility 33% of the score. Complete independence in P-ADL, I-ADL, and mobility resulted in a score of 100%. The patient was instructed on how to complete the FRS. The investigators assisted if the patient could not read and/or complete the form independently.

Quality of life (QoL) was assessed by the Danish version of EQ-5D-5L [31, 32]. Permission to use the tool was obtained from EuroQol Research Foundation. The EQ-5D-5L includes five elements: movement, personal care, usual activities, pain/discomfort, and anxiety/depression, with five response categories: no problems, slight problems, moderate problems, severe problems, and extreme problems/unable to perform. The converted scores of our patients ranged from 1.000 to − 0.624, with 1.000 being the best [33]. In addition to the questionnaire, the assessment of QoL consisted of a vertical visual analog scale (VAS) with scores from 1 to 100, where the endpoints were “the worst health you can imagine” and “the best health you can imagine,” respectively. Patients were asked to complete the questionnaire and VAS according to their health on the day of measurement. Investigators assisted if the patient could not read and complete the form independently.

### Statistical analysis

Although this was a pilot trial, we decided to make a power calculation to assess the feasibility of recruitment and patient acceptability. Prior to the study, we conducted a retrospective, 6-month analysis on geriatric patients meeting the study inclusion criteria and discharged from the geriatric ward at the study hospital. The retrospective analysis showed that 20% were readmitted within 30 days. On the basis of this retrospective analysis, the power calculation resulted in 20 patients being needed in each group in order to obtain a clinically relevant difference of 25% readmission frequency with 80% power, 5% significance level, and 20% dropout rate. Non-parametric statistical tests, median, and range were used, due to the small sample size and skew distributions. The Mann-Whitney *U* test for unpaired data, Wilcoxon signed-rank test for paired data, Fisher’s exact test for categorical data, and intention-to-treat analyses were performed on readmission rate and mortality. Per-protocol analyses were performed for weight, achievement of protein and energy requirements, HGS, 30-SCS, QoL, and FRS. A post hoc analysis was performed to investigate whether readmitted patients differed from not readmitted. No adjustment for multiple testing occurred. All statistical tests were conducted in Excel and GraphPad Prism 7.

## Results

**Fig. 1:**
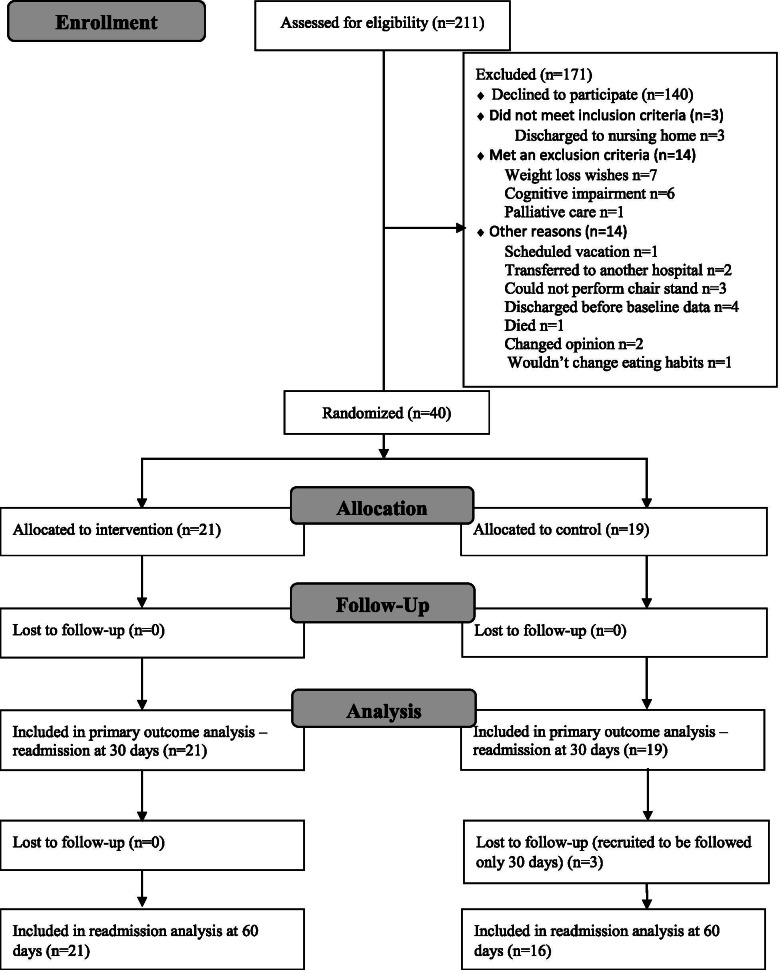
Consort diagram showing the enrollment of patients and numbers of patients included in the analysis for readmission and secondary outcome at 30 days. Two patients in the control group stopped in the trial before 30 days but gave permission for the collection of data regarding readmission

Figure 1 shows the patient flow for readmission and secondary data at 30 days follow-up. The patients were recruited from February to May 2018. In this period, 649 patients aged 65 or older were admitted to the hospital, and 211 of these patients met the study inclusion criteria. Forty of these 211 patients were recruited to the study. There was no difference in age and gender between patients who accepted and those who refused to participate in the study (data not shown). Due to time constraints, 60 days follow-up was not conducted for 7 patients in the intervention group and for 3 patients in the control group. Consequently, data on readmission/mortality were collected for the whole intervention group (*n* = 21), but only part of the control group (*n* = 16) due to three patients in the control group who received dietary guidance after completion of measurements at 30 days. Data after 60 days for the remaining secondary data was only available for 9 and 11 patients in the intervention and control groups, respectively, due to lost to follow-up.

The baseline characteristics are summarized in Table 1. No differences were found between the intervention and control groups. At baseline, only 29% of the patients in the intervention and 32% of the control group met at least 75% of their protein requirements. An equal number of patients in both groups were supplied with a rehabilitation plan by the hospital on discharge. There was high variability in the range of FRS in both groups, i.e., some of the patients had very low functional capacity while others had reasonable functional capacity. Half of the geriatric patients could not accomplish the 30-SCS without using the armrests due to low functional capacity.

Patient acceptance of the intervention was high, with 100% compliance to home visits. All patients not readmitted to the hospital received the first home visit (*n* = 20) and second home visit (*n* = 15).

**Fig. 2:**
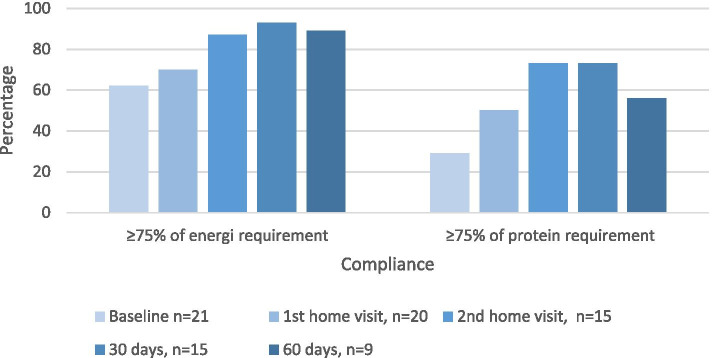
The intervention group’s compliance to achieving a least 75% of energy and protein requirements at baseline, 1st and 2nd home visits, and 30 and 60 days follow-up

The compliance (Fig. 2) to achieving a least 75% of energy requirements increased throughout the trial from baseline (62%) to 30 days follow-up (93%), with a minor decrease at 60 days follow-up (89%). Compliance to achieving a least 75% of protein requirements increased from baseline (29%) to second home visit (73%), stabilizing at 30 days follow-up and decreasing at 60 days follow-up (56%).

Table 2 shows the primary outcome with no difference between the groups in readmission rate within 30 days of discharge from the hospital. No patients were admitted with a new diagnosis. One of the readmitted patients in the intervention group died within 30 days. Two patients in the control group were readmitted within 60 days, and no differences in readmission frequency were found between the groups. No patients were admitted with a new diagnosis.

**Table 2:** Primary outcome: readmission frequency within 30 days—patients had the same hospitalization diagnosis at baseline and readmission

	Intervention, *n* = 21	Control, *n* = 19	*p*-value
Readmission frequency within 30 days, *n* (%)	6 (29)	2 (11)	0.24^a,b^

^a^ITT-analysis

^b^Fisher’s exact test

Five patients were readmitted within the first week after discharge (four from the intervention group, one control). A post hoc analysis for the two groups combined was performed to investigate if the risk factors measured at baseline differed between readmitted and not readmitted. The risk factors assessed were age, BMI, living alone, nutritional risk, length of hospital stay, admissions the year immediately prior to inclusion, and ≤ 75% of energy and protein requirements. Factors with significant differences are shown in Table 3.

**Table 3:** Risk factors for readmission measured at baseline. Only significant differences between the groups are shown

	30 days			60 days		
	Readmitted, ***n*** = 8	Not readmitted, ***n*** = 32	***p***-value	Readmitted, ***n*** = 10	Not readmitted, ***n*** = 30	***p***-value
**Admissions the year prior to inclusion, numbers, median (range)**	4 (0–8)	0 (0–4)	**0.01**	4 (0–8)	0 (0–4)	**0.001**
**Hospital, length of stay, days, median (range)**	9 (5–16)	5 (2–29)	**0.02**	9 (3–16)	5 (2–29)	**0.05**

ITT analysis: Mann-Whitney *U* tests

Table 4 shows the impact of the individual nutritional intervention on secondary outcomes. The individual nutritional intervention had a positive impact on achieving 75% of energy requirements at 30 days after discharge from hospital in the intervention group compared to the control group (93% vs 47%, *p* = 0.01). There was a positive change in HGS from baseline to 60 days in the control group (3 (− 5–11), *p* = 0.05) and in FRS total from baseline to 60 days in the intervention group (3 (0–28), *p* = 0.02). There were otherwise no differences within or between the groups in weight change, HGS, 30-SCS, FRS, or QoL.

**Table 4:** Nutritional status, muscle strength, functional capacity, and quality of life measured at 30 and 60 days after discharge. The *p*-value in column 1 (read horizontally) is for paired data, and the *p*-value in column 4 and 7 is for unparied

	30 days			60 days		
	Intervention, ***n*** = 15	Control, ***n*** = 15	***p***-value^**2**^	Intervention, ***n*** = 9	Control, ***n*** = 11	***p***-value^**2**^
**Nutritional status**						
**Weight development and maintained or increased weight**						
**kg, median (range)**	**74.7 (55.4–105.3)**	**74.0 (52.2–109.1)**	**0.54**	**74.9 (55.7–84.2)**	**74.5 (53.1–101.1)**	**0.66**
**△ from baseline, kg, median (range)**	**− 1.0 (− 4.6–3.0)**	**0.3 (− 4.9–7.1)**	**0.31**	**0.3 (− 3.3–3.3)**	**− 0.6 (− 4.2–3.5)**	**0.33**
***p*-value**^**1**^	**0.08**^**2**^	**0.73**^**2**^		**0.88**^**2**^	**0.77**^**2**^	
**Maintained/increased weight,** ***n*** **(%)**	**6 (40%)**	**8 (53%)**	**0.71**^**1**^	**5 (56%)**	**4 (36%)**	**0.65**^**1**^
**Achievement of ≥ 75% of energy requirement**						
**kJ/day, median (range)**	**7700 (5900–11,600)**	**5900 (4400–9900)**	**0.08**	**7600 (5700–11,900)**	**6500 (3100–10,200)**	**0.26**
**Baseline ≥ 75% of requirements,** ***n*** **(%)**	**8 (53%)**	**7 (47%)**	**> 0.99**^**1**^	**5 (56%)**	**5 (47%)**	**> 0.99**^**1**^
**≥ 75% of requirements,** ***n*** **(%)**	**14 (93%)**	**7 (47%)**	**0.01**^**1**^	**8 (89%)**	**6 (55%)**	**0.16**^**1**^
***p*-value**^**1**^	**0.04**^**1**^	**> 0.99**^**1**^		**0.29**^**1**^	**> 0.99**^**1**^	
**Achievement of ≥ 75% of protein requirement**						
**g/day, median (range)**	**76 (57–107)**	**56 (42–106)**	**0.01**	**76 (49–81)**	**64 (42–113)**	**0.57**
**Baseline ≥ 75% of requirements,** ***n*** **(%)**	**3 (20%)**	**5 (33%)**	**0.68**^**1**^	**2 (22%)**	**4 (36%)**	**0.62**^**1**^
**≥ 75% of requirement,** ***n*** **(%)**	**11 (73%)**	**6 (40%)**	**0.14**^**1**^	**5 (56%)**	**2 (18%)**	**0.16**^**1**^
***p*-value**^**1**^	**0.01**^**1**^	**> 0.99**^**1**^		**0.33**^**1**^	**0.64**^**1**^	
**Muscle strength**						
**Change and increase in handgrip strength (HGS)**						
**kg, median (range)**	**20 (10–46)**	**24 (20–48)**	**0.63**	**20 (11–42)**	**22 (8–48)**	**0.56**
**△ from baseline, kg, median (range)**	**2 (− 4–6)**	**2 (− 4–11)**	**0.52**	**1 (− 3–7)**	**3 (− 5–11)**	**0.38**
***p*-value**^**1**^	**0.20**^**2**^	**0.06**^**2**^		**0.26**^**2**^	**0.05**^**2**^	
**Increased HGS,** ***n*** **(%)**	**8 (53%)**	**10 (67%)**	**0.71**^**1**^	**6 (67%)**	**8 (73%)**	**> 0.99**^**1**^
**Change and increased in 30-s chair stand (30-SCS)**^**a**^						
**30-SCS (WOA), stand ups, median (range)**	**10 (5–11)**	**9 (4–14)**	**0.98**	**9 (5–13)**	**10.5 (2–15)**	**0.68**
**30-SCS (WA), stand ups, median (range)**	**5 (4–8)**	**8.5 (7–13)**	**0.11**	**7 (4–8)**	**6 (5–7)**	**0.90**
**30-SCS from baseline,** ***n*** **(%)**	**11 (79%)**	**13 (93%)**	**0.60**^**1**^	**8 (89%)**	**9 (90%)**	**> 0.99**^**1**^
**Functional capacity**						
**Change in Functional Recovery Score (FRS)**						
**FRS, total**^**b**^	**86 (31–100)**	**89 (33–100)**	**0.56**	**82 (43–100)**	**95 (50–100)**	**0.41**
**△ FRS from baseline**	**1 (− 6–34)**	**2 (− 25–32)**	**0.55**	**3 (0–28)**	**2 (− 8–14)**	**0.59**
***p*-value**^**1**^	**0.16**^**2**^	**0.06**^**2**^		**0.02**^**2**^	**0.08**^**2**^	
**P-ADL**	**44 (25–44)**	**44 (22–44)**	**0.35**	**41 (25–44)**	**44 (2–39)**	**0.12**
**△ P-ADL from baseline**	**0 (− 3–17)**	**0 (− 17–11)**	**0.80**	**0 (− 6–8)**	**0 (− 17–5)**	**0.60**
***p*-value**^**1**^	**0.66**^**2**^	**0.50**^**2**^		**0.38**^**2**^	**0.81**^**2**^	
**I-ADL**	**19 (6–23)**	**18 (2–23)**	**0.94**	**16 (4–23)**	**20 (3–23)**	**0.81**
**△ I-ADL from baseline**	**1 (− 8–6)**	**0 (− 12–13)**	**0.64**	**2 (− 6–4)**	**0 (− 8–4)**	**0.24**
***p*-value**^**1**^	**0.26**^**2**^	**0.70**^**2**^		**0.30**^**2**^	**0.83**^**2**^	
**Mobility**	**25 (0–33)**	**25 (8–33)**	**0.64**	**25 (0–33)**	**33 (17–33)**	**0.40**
**△ Mobility from baseline**	**0 (0–17)**	**0 (0–17)**	**0.55**	**0 (0–17)**	**0 (0–17)**	**0.53**
***p*-value**^**1**^	**0.25**^**2**^	**0.06**^**2**^		**0.50**^**2**^	**0.06**^**2**^	
**Quality of life**						
**Change in quality of life (QoL) and visual analog score (VAS)**						
**EQ-5D-5L**^**c**^	**0.726 (0.139–0.859)**	**0.741 (0.169–0.870)**	**0.85**	**0.740 (0.089–0.833)**	**0.755 (0.291–0.838)**	**0.67**
**△ EQ-5D-5L from baseline**	**0.038 (− 0.044–0.164)**	**0.064(− 0.380–0.412)**	**0.49**	**0.075(− 0.104–0.195)**	**0.076 (− 0.201–0.302)**	**0.56**
***p*-value**^**1**^	**0.16**^**2**^	**0.36**^**2**^		**0.36**^**2**^	**0.20**^**2**^	
**VAS**^**d**^	**65 (40–99)**	**70 (30–90)**	**0.48**	**80 (40–99)**	**75 (50–90)**	**0.93**
**△ VAS from baseline**	**5 (− 35–69)**	**2 (− 30–35)**	**0.91**	**8 (− 25–69)**	**15 (− 9–45)**	**0.72**
***p*-value**^**1**^	**0.34**^**2**^	**0.21**^**2**^		**0.38**^**2**^	**0.03**^**2**^	

PP analysis: Mann-Whitney *U* tests

*I-ADL* instrumental activities of daily living, *P-ADL* personal activity of daily living

^1^Fisher’s exact test

^2^Wilcoxon

^a^Completion of measurement: 30 days—intervention: *n* = 9/5 (WOA/WA), control: *n* = 10/4 (WOA/WA); 60 days—intervention: *n* = 6/3 (WOA/WA), control: *n* = 8/2 (WOA/WA). *WA* with armrest, *WOA* without armrest

^b^FRS total score 0––100 (100 is best)

^c^EQ-5D-5L score 1.000 to − 0.624 (1.000 is best)

^d^VAS score 1–100 (100 best health)

## Discussion

The main findings of this pilot study are that (1) recruitment is feasible, (2) patient acceptance of the intervention is high, and (3) an individual nutritional intervention by educated nutritionists performed at the hospital on the day of discharge, and subsequently in the home of geriatric patients, has a positive effect on nutritional status, but no effect on readmission rate.

Recruitment was feasible, though the acceptance rate was lower than would be expected on the basis of comparable studies, i.e., 22% compared to 37–70% in similar studies [8, 9, 19, 20]. The participants had many diagnoses and a high intake of medicine, and several had multiple hospital admissions in the year before inclusion. The geriatric patients who participated in this pilot study were therefore not healthier or stronger than those who declined to participate. The risk of selection bias is therefore small. Ninety-one percent of the individuals canvassed by the hospital staff agreed to talk to the researchers responsible, while only 22% of these were included in the trial. The main reason given for unwillingness to participate was exhaustion. It is our assessment that a postponed contact with the patient may decrease compliance to the nutritional plan especially concerning the protein intake. A choice between contact by phone and a home visit in the first week after discharge may increase the acceptance rate.

Those who did accept had 100% compliance to the home visits, which is in line with previous studies by Beck et al. [8, 19]. One concern was whether patients’ ability to comprehend the nutritional guidance at the discharge interview and to follow the nutritional plan when they returned home might be compromised by exhaustion. The increase in protein and energy intake found at the first home visit showed that the patients did comply with the nutritional plan, but not enough to achieve a least 75% of requirements. It took 3 weeks and two home visits before most of the patients complied to at least 75% of protein and energy requirements. Compared to baseline, significantly more patients in the intervention group than in the control group achieved 75% of their energy (*p* = 0.04) and protein (*p* = 0.01) requirements at 30 days. The high compliance was due to the simple nutritional plan based on the patients eating habits and preferences, and avoiding oral nutritional supplements if possible. One reason for the delay in compliance was spouses and relatives filling refrigerators with non-protein and non-energy–dense food. Another reason was that it takes time to change diet habits. It is therefore our assessment that in a larger RCT, it would be necessary to involve spouses, relatives, and/or homecare before discharge from the hospital and to make an early home visit/phone call in order to ensure rapid and sustained compliance.

Protein intake decreased after completion of the intervention, from 73% at 30 days to 56% at 60 days follow-up. Protein intake was already low during hospitalization, with only 29% (intervention) and 32% (control) receiving at least 75% of requirements. There was no significant difference between the groups in achieving at least 75% of the energy intake at baseline (62% in the intervention group and 42% in the control group). The difference was much higher at 30 days follow-up, when significantly more patients in the intervention group achieved their energy requirement (93%) compared to the control group (47%) (*p* = 0.01). Seventy-three percent in the intervention group met their protein requirement compared to 40% in the control group within 30 days (not significant). This suggests that it is a challenge for the geriatric patient to meet protein requirements during hospitalization and to maintain a high intake of protein after a 4-week intervention. Beck et al. [8, 19] found that a 12-week individual nutritional intervention significantly increased energy [8] and protein [8, 19] intake in the intervention group compared to the control group. A larger RCT with at least 12 weeks of nutritional intervention is needed to investigate the possibility of effecting a lasting dietary change.

The nutritional intervention had no effect on the readmission rate. Two other studies with comparable interventions found the opposite. Lindegaard et al. [9] found significantly fewer readmissions in the intervention group (11% vs 25%, *p* = 0.03). Energy and protein intake was not measured, so the causality of the individual nutrition intervention in the reduction in readmission frequency could not be argued. Beck et al. (2015) [8] found no difference in readmission (15% intervention vs 19% control), even though the intervention group had a higher intake of energy and protein. The populations in the two studies differed from that in our study, as the patients were older and at nutritional risk. In addition, the patients in the study by Lindegaard et al. [10] lived alone. Other differences were the number of patients receiving daily home care and single vs multidisciplinary intervention. The heterogeneity could well be responsible for the difference in the results.

With four readmissions during the first week, it could be questioned whether the nutritional intervention had any chance to be effective. The post hoc analysis showed that the readmitted patients had significantly longer hospital stays at inclusion (*p* = 0.02) and had significantly more admissions the year before inclusion (*p* = 0.01) compared to those not readmitted. This is not surprising, as a longer hospital stay increases muscle atrophy, prolongs the reconstruction period of muscle mass, and increases the period of rehabilitation [34]. Similar results were found in a cohort study by Alley et al. [3], where a minimum of 8 days of hospitalization within 1 year was associated with a reduction in weight, fat mass, fat-free mass, and muscle strength compared with those not admitted [3]. More admissions within a year and longer hospital stays will most likely escalate the reduction in functional capacity.

Seventy-five percent of the readmitted patients were at nutritional risk at discharge compared to 47% of the not readmitted. This is in line with a recent prospective observational study by Sharma et al. [35] including 297 older medical patients. Malnutrition at hospitalization was associated with a significantly higher risk of readmission or death within 7 days (OR 4.57, 95% CI 1.69 to 12.37, *p* < 0.001) and within 8–180 days after discharge from hospital (OR 1.98, 95% Cl 1.19 to 3.28, *p* = 0.01). Seventy-five percent of the readmitted lived alone, compared to 38% of the not readmitted. It can be hypothesized that geriatric patients with the highest risk of readmission are those living alone, at nutritional risk, admitted to the hospital several times during the past year, and with a longer hospital stay, and this cannot be compensated by nutritional counseling as the only intervention at discharge.

There were no further readmissions in the intervention group, but two in the control group within 60 days (29% vs 21%). Four similar studies with home visits and nutritional counseling have investigated hospital admission frequency at 3 and 6 months [8, 9, 19, 20]. Only one study [9] found significantly fewer readmissions in the intervention group compared to the control group (18% vs 39%, *p* < 0.01) at 3 months. In contrast to our study, Beck et al. [8] did not exclude patients at readmission, and even though the nutritional intervention did not reduce readmission frequency, it did reduce the number of hospitalizations within 6 months. This is a gain for the geriatric patient [3], and it reduces health care costs [36]. The primary outcome in a future definitive RCT might therefore be the number of hospitalizations instead of readmission at 30 days, which would mean that the patients should remain in the study after readmission. An important patient-related secondary outcome should be the length of hospitalization.

The intervention in this study was nutrition only and not physical activity. A third of the patients in both groups received a rehabilitation plan provided by the hospital. The patients were fragile when they arrived home and did not activate the plan until weeks after discharge. The lack of significant results indicates that nutrition alone cannot improve ADL and mobility. Including physical activity in a larger RCT may have a positive effect on relevant outputs such as functional capability, ADL, muscle strength, quality of life, and number and duration of hospitalizations.

The major strength of the present study was the high compliance to home visits and to the individual nutritional plan, indicating that the geriatric patients were content with the individual nutritional intervention. One of the other strengths were that the primary outcome was without bias, as randomization after baseline reduced the risk for confounding, or selection and performance bias in the baseline data.

The lack of blinding was a weakness in this study, increasing the risk of information bias. On the other hand, this design with the same person observing and following up is similar to clinical practice. Recall and information bias was a risk in the 24-h dietary interview due to patients’ lapses of memory and to the lack of neutrality by the investigators. Other comparable studies have used 4 days dietary records, which seem to be more precise on the premise of high compliance to filling in the records. The consideration in this study was that it would be too large a burden for the geriatric patients. At the beginning of the study, the patients did not have the energy or mental capacity to keep a dietary record for 4 days, but at 30 and 60 days follow-up, it would have been feasible, with phone calls to patients reminding them to record their dietary intake. At baseline, a 2–4-day diet and fluid registration at the hospital, depending on the length of hospitalization, could be the solution. Compliance should be investigated before deciding what to use in a full-scale RCT.

The individual nutritional intervention was not time-consuming. A home visit, including preparation, transport, and data processing, took approximately 2 h. The intervention was well received by the geriatric patients, and no side effects were observed. It should be possible to apply the intervention in a future large-scale RCT, even with additional home visits and phone calls.

Qualified, individual guidance concerning nutrition led to a better intake of both energy and protein but did not influence other patient-relevant outcomes. This, together with the findings of other studies, suggests that nutrition alone cannot determine the patient’s health after discharge from the hospital and that it must be considered together with other factors, such as physical activity, social network, and severity of disease, in the design of a full-scale RCT.

In conclusion, this pilot study shows that it is feasible to recruit geriatric patients to a nutrition intervention on discharge from the hospital and that there is high compliance to the intervention. Our results justify that further investigation and a full-scale RCT should consist of a multidisciplinary intervention of at least 12 weeks, with a primary outcome of the number of readmissions within 6–12 months of discharge from the hospital, and duration of hospitalizations as the secondary outcome.

## Supplementary Information

**Additional file 1.** CONSORT 2010 checklist of information to include when reporting a pilot or feasibility trial.

## Data Availability

The datasets used and/or analyzed during the current study are available from the corresponding author on reasonable request.

## Abbreviations

ADL: Activities of daily living
BMR: Basic metabolic rate
CORD: Chronic obstructive pulmonary disease
FRS: Functional Recovery Score
HGS: Handgrip strength
I-ADL: Instrumental activity of daily living
PA: Physical activity
P-ADL: Personal activities of daily living
PAL: Physical activity level
QoL: Quality of life
VAS: Visual analog scale
30-SCS: 30-s chair stand
